# Systematic Screening of Chemokines to Identify Candidates to Model and Create Ectopic Lymph Node Structures for Cancer Immunotherapy

**DOI:** 10.1038/s41598-017-15924-2

**Published:** 2017-11-22

**Authors:** Yohsuke Yagawa, Mark Robertson-Tessi, Susan L. Zhou, Alexander R. A. Anderson, James J. Mulé, Adam W. Mailloux

**Affiliations:** 1 Department of Immunology, Moffitt Cancer Center, 12902 Magnolia Drive, Tampa, FL 33612 USA; 2 Department of Integrated Mathematical Oncology, Moffitt Cancer Center, 12902 Magnolia Drive, Tampa, FL 33612 USA; 3 Cutaneous Oncology Program, Moffitt Cancer Center, 12902 Magnolia Drive, Tampa, FL 33612 USA

## Abstract

The induction of ectopic lymph node structures (ELNs) holds great promise to augment immunotherapy against multiple cancers including metastatic melanoma, in which ELN formation has been associated with a unique immune-related gene expression signature composed of distinct chemokines. To investigate the therapeutic potential of ELNs induction, preclinical models of ELNs are needed for interrogation of these chemokines. Computational models provide a non-invasive, cost-effective method to investigate leukocyte trafficking in the tumor microenvironment, but parameterizing such models is difficult due to differing assay conditions and contexts among the literature. To better achieve this, we systematically performed microchemotaxis assays on purified immune subsets including human pan-T cells, CD4^+^ T cells, CD8^+^ T cells, B cells, and NK cells, with 49 recombinant chemokines using a singular technique, and standardized conditions resulting in a dataset representing 238 assays. We then outline a groundwork computational model that can simulate cellular migration in the tumor microenvironment in response to a chemoattractant gradient created from stromal, lymphoid, or antigen presenting cell interactions. The resulting model can then be parameterized with standardized data, such as the dataset presented here, and demonstrates how a computational approach can help elucidate developing ELNs and their impact on tumor progression.

## Introduction

Despite advances in immunotherapy and other treatment options, melanoma remains an increasing concern for caregivers, with over 60,000 new diagnoses of invasive melanoma per year in the United States^1^, and over 112,000 cases projected per year by 2030^2^. If detected early, surgical resection offers the best outcome and can often be curative. However, once the disease becomes metastatic, the prognostic outlook is bleak with only 16% of patients surviving 5 years^3^. For metastatic disease, immunotherapy can offer a handful of attractive options, which display potent but often incomplete clinical responses^4–6^. The use of cytokines and the more recent implementation of antibodies against immune checkpoint receptors CTLA-4 or PD-1 all display dramatic and durable clinical responses in a minority of patients^7^. Juxtaposed to this group of biologics is the adoptive transfer of autologous tumor-infiltrating lymphocytes (TIL) expanded *ex vivo* from patient tumors. TIL therapy was first pioneered at the NCI Surgery Branch^8,9^, and is now available at several institutions in the U.S. and abroad^10–13^. When combined with lympho-depleting, non-myeloablative chemotherapy prior to adoptive transfer, TIL therapy can display clinical response rates approaching 50%^14,15^.

The initial presence of lymphocytes in the tumor microenvironment is presumptive to the success of any immunotherapy. The prognostic association of immune infiltrate in metastatic melanoma was at first contested, with some reports that TIL presence serves as an independent prognostic indicator^16–18^, and others reporting no association with clinical outcome or lacking independence as a prognostic factor^19–21^. More detailed investigation suggests that taking into account the activation state or proliferation rate of TIL can better indicate positive prognosis^22^. Of importance, recent observations suggest that the presence of tumor-localized, ectopic lymph node structures (ELNs) is associated with better prognosis across a broad spectrum of tumor types including metastatic melanoma^23^, breast cancer^24^, colorectal carcinoma^25^, and non-small cell lung cancer^26,27^. ELNs are highly organized aggregates of leukocytes, often displaying distinct T cell and B cell zones, as well as, in some cases, clearly defined marginal zones with activated antigen presenting cells^28^. Structural features of ELNs, such as the *de novo* generation of lymphatic vessels, can greatly enhance the infiltration of TIL deeper into the tumor parenchyma^29^. Such dissemination away from the vasculature is highly associated with better clinical outcome^30^. While ELNs may be beneficial for the majority of cancer types, this is not universally true. The presence of ELNs serves as a negative prognostic indicator for a few cancer types such as hepatocellular carcinoma, and was associated with polarized immune cell subsets or suppressed immune response^31^ demonstrating a clear dichotomy based on different microenvironments. Taken together, the organization, activation state, and polarization of the microenvironment appear just as important as the number of TIL.

Looking forward, the ability to induce or construct ELNs with anti-tumor activity holds great promise to help recruit TIL to the tumor microenvironment and enhance their anti-tumor activity, particularly in solid tumors devoid of these structures. To help develop such a strategy requires the creation of sound preclinical models in which to study ELNs formation. The localization of lymphocytes is largely governed by networks of chemokines, which guide their trafficking to different parts of the body at different stages of development, maturation, and activation. In similar fashion to the trafficking in a conventional peripheral lymph node, lymphocyte involvement in ELNs likely depends on a network of chemokines produced by resident stroma or resident leukocytes such as dendritic cells. Indeed, our previous work identified a tumor gene expression signature associated with the presence of ELNs in certain human solid tumors that encodes for 12 distinct chemokines^23–25,32^.

We are interested in employing these chemokines as leads to construct or induce ELNs in the solid tumor microenvironment with the intent to potentially enhance immunotherapies, particularly in those devoid of such structures. To achieve this goal will first require a series of fundamental biologic studies and modeling. It is known that chemokines and their cognate receptors have been extensively studied, but differing methodologies and sources of responding cells have made comparing chemokine/chemokine receptor axes across publications difficult to interpret. A dataset reporting chemoattractive potential using a single methodology as well as a similar cell isolation technique, migration time, and chemokine concentration range would greatly enhance the parameterization of *in silico* ELNs models, and inform preclinical *in vivo* murine models investigating ELN formation and function. Here, we have used a conventional transwell migration assay to first catalogue the chemotactic index (CI) of 48 recombinant murine chemokines on resting pan T cells, CD4^+^ T cells, CD8^+^ T cells, B cells, and NK cells immunomagnetically isolated from normal C57BL/6 spleen, including the 12 chemokines associated with ELNs formation in humans. We also catalogue the CI for select chemokines for activated B cells and T cells. Using this database, the CI of different chemokines can be directly compared, and may serve as a valuable tool for the parameterization of preclinical animal models. In addition, we introduce a groundwork mathematical model able to make use of standardized chemoattraction data that can serve as a basic infrastructure for more complex models in the future. The model makes use of reticular fibroblast cells (RFC) as a stromal source of chemokine production^28^, antigen presenting cells as an activating source for RFC and chemokine production, and generalized T cell and B cell populations responding to chemokine gradients^32,33^.

## Materials and Methods

### Animals

Female C57BL/6 mice were purchased from Harlan Laboratories (Indianapolis, IN). Mice were bred and maintained at the Animal Maintenance Facility at the H. Lee Moffitt Cancer Center and Research Institute (Tampa, FL) for at least 1 week prior to use, and were age-matched at 8 weeks or older before their usage in experiments. All mice were handled and treated in accordance with the institutional guidelines established by the animal review board for animal care at the H. Lee Moffitt Cancer Center and all experimental protocols were approved by the Institutional Animal Care and Use Committee (IACUC) at the University of South Florida.

### Culture Medium

Complete medium (CM) consisted of RPMI 1640 medium with 10% heat-inactivated fetal bovine serum, 1 μM sodium pyruvate, 0.1 mM nonessential amino acids, 2 mM fresh L-glutamine, 100 μg/mL streptomycin, 100 U/mL penicillin, 50 μg/mL gentamicin, 0.5 µg/mL fungizone (all from Life Technologies, Rockville, MD), and 0.05 mM β-mercaptoethanol (Sigma-Aldrich, St. Louis, MO).

### Chemokines

Recombinant murine CCL2, 3, 4, 5, 6, 7, 8, 9/10, 11, 12, 19, 20, 21, 22, 24, 27, 28, CXCL1, 2, 4, 5, 9, 10, 11, 12 SDF1β, 13, 15, and 16 were purchased from PeproTech (Rocky Hill, NJ), and CCL17, 25, CXCL3, 17, CX3CL1, XCL1 and Chemerin were obtained from R&D Systems (Minneapolis, MN). In some cases, murine chemokines were not available, and human chemokines were used due to their known cross-species activity. Human CCL16, 18, CXCL6 and 8 were purchased from PeproTech, XCL2 was from Life Technologies (Carlsbad, CA) and CCL1, 13, 14, 15, 23, 26, CXCL7 and 14 were provided by ChemoCentryx (Mountain View, CA).

### Isolation of Lymphocyte Populations

Spleens and the superficial inguinal, axillary, lateral axillary, mesenteric and cervical lymph nodes of mice were removed under sterile conditions and mechanically dissociated to prepare single-cell suspension with a 100-µm nylon mesh. Erythrocytes were lysed with RBC lysing buffer (0.15 M NH_4_Cl, 1 mM KHCO_3_, and 0.1 mM EDTA in sterile water). After incubation for 1 minute, cells were washed in 1x PBS and used for further experiments.

Pan T, CD4^+^ T, CD8^+^ T, B and NK cells were then isolated from splenocyte and lymph node cell suspensions using negative immunomagnetic isolation kits as per the manufacturer’s instructions: Pan T Cell isolation kit II, CD4^+^ T Cell isolation kit II, CD8α^+^ T Cell isolation kit II, B Cell isolation kit and NK isolation kit for mouse (Miltenyi Biotech, Auburn, CA). Isolations were performed using a magnetic cell sorter (autoMACS) (Miltenyi Biotech, Auburn, CA). Pan T and B cell isolation enrichments were greater than 95%, CD4^+^ T and CD8^+^ T cells were greater than 90% and NK cells were greater than 80% as determined by flow cytometric analysis. See supplemental Fig. 1.

### Flow Cytometry

Cells were washed with flow buffer (0.01% NaN_3_, 2% FBS in PBS) and Fcγ III/II receptor blocking was performed for B and NK cells by purified anti-mouse CD16/32 Fc blocking antibody (BD Biosciences, San Diego, CA). After incubation at 4 °C for 5 minutes, appropriate antibodies (1 µg/1 × 10^6^ cells) for each cell marker were added to each sample and placed at 4 °C for 30 minutes. After washing with flow buffer, cells were fixed with 1% paraformaldehyde. Data acquisition was performed on FACSCalibur (Becton Dickinson, Mountain View, CA) and data analysis was done using FlowJo (Treestar, Ashland, OR). CD3^+^ CD19^−^, CD3^+^ CD4^+^, CD3^+^ CD8^+^, CD19^+^ CD3^−^ and NK1.1^+^ CD3^−^ cells were measured for Pan T, CD4^+^ T, CD8^+^ T, B and NK cell, respectively (all antibodies and isotype controls from BD Biosciences). For staining chemokine receptors, cells were incubated with anti-CCR7, -CXCR4 (BD Biosciences) or -CXCR5 antibodies (eBiosciences, San Diego, CA) for 40 minutes at room temperature.

### Microchemotaxis Assay

Each chemokine was added to the lower chamber of a 24-well transwell (Costar, Cambridge, MA) at indicated concentrations in 600 μL of CM, and incubated at 37 °C in a humidified incubator with 5% CO_2_ for 30 minutes. Respective lymphocyte populations were resuspended at 1 × 10^7^ cells/mL in CM, and 100 μL (1.0 × 10^6^ cells) were seeded into 6.5-mm 24-well transwell inserts (Costar, Cambridge, MA), and allowed to incubate at 37 °C for 10 minutes. After pre-incubations, the upper chambers were moved into lower wells to start assay. To determine the actual input amount, 100 μL of cell suspension was added to 500 μL of CM and put into the lower wells directly and incubated without upper chambers. After 3 hour incubation at 37 °C, the upper chambers were removed and the migrating cells were retrieved. Cells were stained with the appropriate antibodies and added 2 × 10^5^ polystyrene beads (Bangs Laboratories, Fishers, IN). Twenty thousand beads were then counted flow cytometrically. Baseline chemokinesis was determined by the fraction of cells moving into the lower chamber in control wells containing no chemotactic agent, and is expressed as a percent of seeded cells. The chemotactic index (CI) is calculated as the fraction of cells migrating in response to the chemotactic agent normalized to the chemokinesis background such that CI = (fraction of cells migrating to condition)/(fraction of cells migrating to control media). Transwell migration through 3 μm, 5 μm, and 8 μm pore sizes was compared (Supplemental Fig. 2), and the 5 μm pore size was selected for use. Data reported are representative of at least two experiments.

### Activation Assay

Prior to CD4^+^ and CD8^+^ T cell activation, the culture plate was coated with 5 μg/mL of anti-CD3 and 1 μg/mL of anti-CD28 antibodies (BD Biosciences). T cells were resuspended to 1 × 10^6^ cells/mL in CM and cultured at 37 °C for 5 days in the presence of 60 IU/mL of IL-2 (Prometheus, San Diego, CA). For B cell activation, B cells were resuspended in RPMI 1640/1% FBS containing 10 μg/mL LPS (Sigma-Aldrich) and cultured for 3 days. Analysis was performed on CD4^+^CD25^+^, CD8^+^CD25^+^ and CD19^+^CD69^+^ cells for activated CD4^+^ T, CD8^+^ T and B cells, respectively (Supplemental Fig. 3A–C).

### Enzyme-linked Immunosorbent Assay (ELISA)

ELISA was carried out to evaluate T and B cell activation (Supplemental Fig. 3D). After supernatants were harvested at day 5 in the T cell activation assay or at day 3 in the B cell activation assay, IFNγ and IgM were measured, respectively. Mouse IFNγ ELISA Kit II (BD Biosciences) and Mouse IgG ELISA Quantitation Set (Bethyl laboratories, Montgomery, TX) were used in this experiment according to the manufacturer’s instructions.

### Statistical Analysis

Unpaired student t-test was performed for each chemokine concentration versus control wells containing no chemokine using GraphPad Prism 5.04 software (GraphPad Software, La Jolla, CA). Microchemotaxis assays were performed on individually isolated/activated wells for each cell population (n = 4).

### Mathematical Model

A cellular automaton model was developed to investigate the effect of ELNs on the organization and effectiveness of the immune response to a nearby tumor. The mathematical model is simulated in two dimensions and contains a tumor, a patch of fixed RFC, and four types of motile cells that move in a continuous domain (i.e., off-lattice): inactive T cells, activated T cells, resting antigen presenting cells (APC^OFF^), and activated marginal zone antigen presenting cells (APC^M^). The tumor is represented by a circle of radius *R*, where we assume that the radius grows linearly in time in the absence of any immune response, and that the T-cell response causes a reduction in growth rate proportional to the number of tumor-infiltrating lymphocytes (*L*). The equation for tumor growth per time step of the simulation is given by:

1 $${R}_{t+1}={R}_{t}+(g-kL){\rm{\Delta }}t$$

where *g* is the innate tumor growth rate, *k* is the killing rate of TILs, and *Δt* is the time step used in the simulation. Assuming a disease state in which anti-tumor cytotoxicity can occur, increasing numbers of TILs would slow the growth and potentially cause the tumor to regress if *kL* > *g*.

APC^OFF^ are introduced into the tumor area at a constant rate. The new cells are randomly positioned in an annulus centered around the tumor, with inner radius of (*R* − 500 µm) and outer radius of (*R* + 1500 µm). Once in the environment, they move with a combination of an unbiased random walk and a directed walk towards the tumor center, following tumor-produced inflammatory chemokine gradients. When an APC^OFF^ is inside the tumor, the antigen collection process is simulated by converting the cell to an APC^M^, which can present antigen to the T cells. These APC^M^ are also motile, using an unbiased random walk in combination with a directed walk away from the tumor center, to represent the seeking of T cells and/or vasculature for subsequent antigen presentation.

Inactive T cells are added to the simulation with a constant rate, using the same annulus as for APC^OFF^ cells. These cells have an unbiased random walk. When an inactive T cell encounters an APC^M^ (based on the two cells being in proximity to each other at a given time step), the APC^M^ activates the T cell. The APC^M^ can activate a number of T cells before it is removed from the simulation. Active T cells follow a biased random walk in the direction of the tumor. When they cross the tumor boundary as determined by *R*, they become TIL, which affects the tumor killing rate as shown in Eq. 1. They remain TIL for a set period of time, after which they are removed from the simulation, as expired T cells have no further effect on the results.

To model the effect of ELNs, we randomly place a fixed number of RFC in a circular patch situated away from the tumor. Each RFC begins in the “off state” with no secretion of chemokines, and therefore no influence on the movement of any of the APC or T cells. However, when an APC^M^ migrates and comes into “contact” with an RFC (based on the two cells’ proximity to each other), it will activate the RFC, which will in turn start to produce a chemokine gradient. For simplicity here, we model the chemokine gradient produced by an activated RFC as a two-dimensional Gaussian function centered at the cell. The total chemokine gradient for multiple activated RFCs is the sum of these Gaussians. As more RFC are activated, the chemokine signal becomes stronger. This gradient affects two of the motile cells, APC^M^ and inactive T cells. For both of these cell types, when they detect the presence of chemokine gradient produced by RFC, movement is biased toward the ELNs patch. The bias scales with the strength of the gradient. We vary the number of RFC in different simulations to investigate this effect.

The random walks in the model were implemented as follows: the motile cells in the simulation are off-lattice and therefore can take any value for their position. The maximum travel distance for a cell in a time step is its kinetic speed multiplied by the time step. The algorithm selects a random distance between 0 and this maximal distance, and moves the cell in the direction of a randomly chosen angle. When there is an additional chemotactic term, a second random distance is chosen based on the maximum tactic speed, but the angle is fixed, directed towards the chemokine source (ascending the gradient). In this model, we do not consider spatial occupancy, so cells can get arbitrarily close to each other.

To summarize the model, there are two chemokine gradients, one from the tumor and one from the RFC^M^. APC^OFF^ and active T cells move towards the tumor, and APC^M^ and inactive T cells move away from the tumor. When there are activated RFC^M^, APCM and inactive T cells are both drawn to the ELNs. The simulation is initialized with a small tumor, a patch where RFC^OFF^ cells are placed, and 30 APC^OFF^ and inactive T cells. Parameters used in the model are shown in Table 1, with those labeled as ‘model specific’ refer to parameters that are highly variable in practice (e.g., tumor size, ELN-tumor distance, influx rate of immune cells); values were chosen that were in line with biologically reasonable ranges.

**Table 1:** Parameters and initial conditions for the mathematical model.

Name	Value	Units	Reference
Growth rate of Tumor (*g*)	0.2	μm/min	41
Tumor death rate due to TIL (*k*)	0.0015	μm/min/cell	42
Kinetic speed of APC^OFF^	12	μm/min	43
Chemotactic speed of APC^OFF^	4	μm/min	43
Kinetic speed of APC^M^	20	μm/min	43
Chemotactic speed of APC^M^	10	μm/min	43
Kinetic speed of T cells	10	μm/min	44,45
Chemotactic speed of T cells	100	μm/min	44,45
Rate of adding new APC^OFF^ to simulation	0.84	per min	model specific
Rate of adding new inactive T cells to simulation	0.1	per min	model specific
Lifespan of a TIL before expiration	1	day	model specific
Max distance between two cells for activation to occur	15	μm	model specific
Initial Conditions:			
Initial radius of the Tumor	200	μm	model specific
Radius of the ELN patch	750	μm	model specific
Distance from tumor to ELNs patch	2000	μm	model specific
Initial T-cell number	30	cells	model specific
Initial APC^OFF^ number	10	cells	model specific

## Results

**Figure 1:**
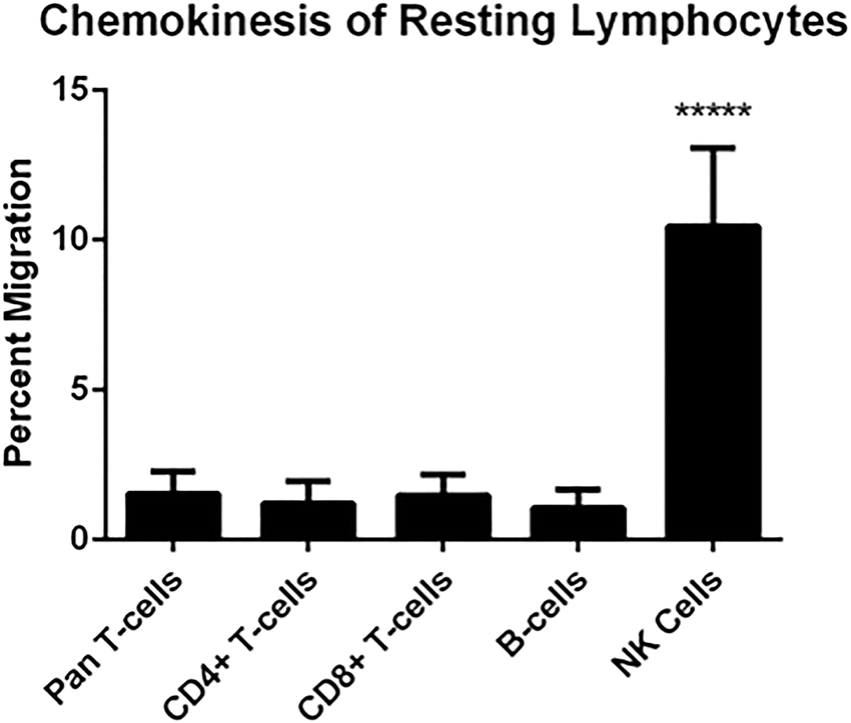
Resting NK cells display higher chemokinesis than other lymphocyte populations. Chemokinesis is reported as the baseline random migration into the lower chamber of a control transwell assay containing no chemoattractants. ****p < 0.0001.

Resting pan T cells, B cells, CD4^+^ T cells, CD8^+^ T cells, or NK cells were immunomagnetically isolated and chemotaxis assays were performed for the 48 chemokines listed in Table 2 at three different concentrations: 10 ng/mL, 100 ng/mL, and 1,000 ng/mL. Non-directional random motility, or chemokinesis, was assessed for each cell population using control wells containing no chemokine gradient. Resting pan T cells, CD4^+^ T cells, CD8^+^ T cells, and B cells all displayed relativity low levels of chemokinesis while NK cells displayed significantly higher non-directional movement (Fig. 1). Directional movement in response to a chemokine concentration gradient was normalized to baseline chemokinesis for each respective cell type, and is reported as a chemotactic index (CI) for each condition. CI was calculated as the fraction of cells that migrated into the lower chamber from the total number of cells seeded in the top chamber for each condition, and then divided by the fraction of cells that migrated into the lower chamber randomly in control wells (i.e. by chemokinesis).

**Table 2:** Chemotactic Index (CI) of Chemokines on lymphocyte Populations.

Chemokine	(ng/ml)	Pan T cell	CD4^+^ T cell	CD8^+^ T cell	B cell	NK cell
CCL1	0	1 ± 0.38	1 ± 0.47	1 ± 0.32	1 ± 0.39	1 ± 0.23
	10	1.02 ± 0.24	1.09 ± 0.17	0.77 ± 0.16	0.94 ± 0.19	1.01 ± 0.24
	100	0.77 ± 0.23	0.78 ± 0.26	0.78 ± 0.25	0.62 ± 0.27	1.01 ± 0.4
	1,000	1.17 ± 0.42	1.28 ± 0.56	0.89 ± 0.29	1.01 ± 0.45	0.92 ± 0.29
CCL2	0	1 ± 0.09	1 ± 0.54	1 ± 0.27	1 ± 0.47	1 ± 0.03
	10	0.96 ± 0.14	1.6 ± 1	1.03 ± 0.3	0.63 ± 0.32	3.78 ± 0.22***
	100	1.2 ± 0.38	0.9 ± 0.4	0.99 ± 0.42	1.24 ± 1.12	3.23 ± 0.25**
	1,000	1 ± 0.09	1.04 ± 0.53	0.97 ± 0.29	0.72 ± 0.54	1.77 ± 0.08**
CCL3	0	1 ± 0.25	1 ± 0.27	1 ± 0.24	1 ± 0.23	1 ± 0.14
	10	0.77 ± 0.17	0.77 ± 0.23	0.75 ± 0.19	0.66 ± 0.21	1.34 ± 0.11
	100	0.86 ± 0.11	0.8 ± 0.11	0.78 ± 0.09	0.68 ± 0.06	1.79 ± 0.25**
	1,000	0.85 ± 0.08	0.86 ± 0.08	0.78 ± 0.05	0.89 ± 0.15	1.38 ± 0.07*
CCL4	0	1 ± 0.23	1 ± 0.29	1 ± 0.33	1 ± 0.26	1 ± 0.09
	10	1.26 ± 0.11	1.33 ± 0.08	1.15 ± 0.07	1.37 ± 0.07	1.35 ± 0.03**
	100	1.3 ± 0.1	1.33 ± 0.12	1.33 ± 0.22	0.75 ± 0.09	1.75 ± 0.11***
	1,000	1.13 ± 0.11	1.24 ± 0.15	1.08 ± 0.03	0.9 ± 0.12	1.42 ± 0.3
CCL5	0	1 ± 0.23	1 ± 0.29	1 ± 0.33	1 ± 0.26	1 ± 0.09
	10	1.25 ± 0.09	1.26 ± 0.16	1.23 ± 0.15	0.91 ± 0.27	1.37 ± 0.03**
	100	1.15 ± 0.08	1.05 ± 0.12	1.3 ± 0.11	0.76 ± 0.13	1.62 ± 0.1**
	1,000	1.22 ± 0.14	1.25 ± 0.17	1.1 ± 0.16	0.93 ± 0.16	1.52 ± 0.11**
CCL6	0	1 ± 0.08	1 ± 0.09	1 ± 0.06	1 ± 0.17	1 ± 0.26
	10	0.91 ± 0.08	0.97 ± 0.14	0.84 ± 0.27	1.82 ± 0.21	0.82 ± 0.17
	100	0.87 ± 0.08	0.73 ± 0.07	0.97 ± 0.06	1.56 ± 0.09	0.91 ± 0.17
	1,000	1.01 ± 0.24	0.96 ± 0.36	1.12 ± 0.24	1.29 ± 0.18	1 ± 0.18
CCL7	0	1 ± 0.57	1 ± 0.69	1 ± 0.55	1 ± 0.56	1 ± 0.14
	10	0.7 ± 0.34	0.65 ± 0.29	0.81 ± 0.49	0.4 ± 0.04	1.36 ± 0.1
	100	0.94 ± 0.56	0.82 ± 0.52	2.51 ± 1.89	0.5 ± 0.06	1.76 ± 0.16*
	1,000	1.35 ± 0.85	0.73 ± 0.36	1.54 ± 1.12	0.67 ± 0.17	1.94 ± 0.05*
CCL8	0	1 ± 0.23	1 ± 0.29	1 ± 0.33	1 ± 0.26	1 ± 0.09
	10	0.96 ± 0.08	1.02 ± 0.15	0.89 ± 0.07	1.26 ± 0.35	0.97 ± 0.11
	100	0.8 ± 0.01	0.79 ± 0.05	0.74 ± 0.09	0.72 ± 0.03	0.97 ± 0.07
	1,000	1.1 ± 0.17	1.06 ± 0.21	1.07 ± 0.25	1.36 ± 0.63	1.05 ± 0.12
CCL9	0	1 ± 0.08	1 ± 0.09	1 ± 0.06	1 ± 0.17	1 ± 0.26
	10	0.91 ± 0.07	0.77 ± 0.11	3.76 ± 1.77	1.96 ± 0.62	1.05 ± 0.13
	100	0.86 ± 0.13	0.71 ± 0.18	0.99 ± 0.36	1.96 ± 0.62	0.93 ± 0.18
	1,000	1.14 ± 0.22	1.21 ± 0.57	1.25 ± 0.63	2.18 ± 0.59*	0.99 ± 0.05
CCL11	0	1 ± 0.08	1 ± 0.09	1 ± 0.06	1 ± 0.17	1 ± 0.26
	10	0.75 ± 0.03	0.77 ± 0.27	0.99 ± 0.35	1.05 ± 0.09	0.88 ± 0.15
	100	0.75 ± 0.15	0.74 ± 0.2	0.89 ± 0.29	1.05 ± 0.09	0.82 ± 0.19
	1,000	1.09 ± 0.4	1.16 ± 0.46	1.29 ± 0.52	2.51 ± 0.56*	1.35 ± 0.4
CCL12	0	1 ± 0.08	1 ± 0.09	1 ± 0.06	1 ± 0.17	1 ± 0.26
	10	0.8 ± 0.06	0.9 ± 0.1	1.06 ± 0.37	2.01 ± 0.2**	0.97 ± 0.22
	100	0.94 ± 0.08	0.99 ± 0.12	1.06 ± 0.37	1.62 ± 0.51	1.51 ± 0.42
	1,000	1.21 ± 0.44	1.26 ± 0.36	1.46 ± 0.67	1.96 ± 0.64	2.54 ± 0.34*
CCL13	0	1 ± 0.08	1 ± 0.07	1 ± 0.04	1 ± 0.18	1 ± 0.05
	10	1.13 ± 0.08	1.09 ± 0.1	1.23 ± 0.02***	0.94 ± 0.07	1.91 ± 0.07****
	100	1.42 ± 0.11	1.3 ± 0.09*	1.72 ± 0.1***	1.18 ± 0.1	2.01 ± 0.06****
	1,000	2.05 ± 0.44	1.72 ± 0.39*	2.88 ± 0.46**	1.18 ± 0.14	1.96 ± 0****
CCL14	0	1 ± 0.08	1 ± 0.18	1 ± 0.04	1 ± 0.18	1 ± 0.05
	10	1.13 ± 0.19	1.2 ± 0.12	0.91 ± 0.12	0.94 ± 0.07	0.92 ± 0.03
	100	1.02 ± 0.27	0.87 ± 0.07	0.83 ± 0.18	1.18 ± 0.1	0.95 ± 0.1
	1,000	1.16 ± 0	1.08 ± 0.1	1.08 ± 0.04	1.18 ± 0.14	1.37 ± 0.23
CCL15	0	1 ± 0.1	1 ± 0.11	1 ± 0.09	1 ± 0.13	1 ± 0.14
	10	0.87 ± 0.04	0.83 ± 0.09	0.87 ± 0.07	0.71 ± 0.09	0.83 ± 0.08
	100	0.89 ± 0.03	0.93 ± 0.07	0.87 ± 0.03	1.09 ± 0.27	1.01 ± 0.01
	1,000	1 ± 0.07	1.04 ± 0.07	0.99 ± 0.17	0.86 ± 0.1	0.88 ± 0.06
CCL16	0	1 ± 0.17	1 ± 0.15	1 ± 0.16	1 ± 0.18	1 ± 0.23
	10	1.84 ± 0.21**	2.04 ± 0.41*	1.68 ± 0.12	1.42 ± 0.55	1.11 ± 0.22
	100	1.48 ± 0.07**	1.53 ± 0.13**	1.42 ± 0.07	1.08 ± 0.06	0.98 ± 0.06
	1,000	1.12 ± 0.05	1.21 ± 0.07	1.09 ± 0.05	0.97 ± 0.45	0.94 ± 0.1
CCL17	0	1 ± 0.38	1 ± 0.47	1 ± 0.32	1 ± 0.39	1 ± 0.23
	10	0.83 ± 0.14	0.86 ± 0.21	0.64 ± 0.21	0.65 ± 0.06	0.91 ± 0.31
	100	1.15 ± 0.36	1.33 ± 0.5	0.8 ± 0.21	0.5 ± 0.21	0.91 ± 0.31
	1,000	0.98 ± 0.38	1.07 ± 0.44	0.74 ± 0.35	0.83 ± 0.32	0.84 ± 0.35
CCL18	0	1 ± 0.25	1 ± 0.27	1 ± 0.24	1 ± 0.23	1 ± 0.14
	10	1.01 ± 0.22	1.1 ± 0.23	1.02 ± 0.24	0.42 ± 0.17	0.87 ± 0.32
	100	1.01 ± 0.13	1.05 ± 0.1	0.97 ± 0.14	0.26 ± 0.06	0.88 ± 0.24
	1,000	0.98 ± 0.06	1.06 ± 0.04	1 ± 0.09	0.36 ± 0.12	0.7 ± 0.04
CCL19	0	1 ± 0.33	1 ± 0.38	1 ± 0.34	1 ± 0.41	ND
	10	5.26 ± 0.65**	9.02 ± 1.05***	1.23 ± 0.19	1.89 ± 0.46	ND
	100	14.19 ± 1.1****	20.6 ± 1.56****	8.69 ± 0.79***	2.21 ± 0.19**	ND
	1,000	27.73 ± 3.11***	28.3 ± 3.45***	33.0 ± 3.28****	2.43 ± 0.71*	ND
CCL20	0	1 ± 0.08	1 ± 0.09	1 ± 0.06	1 ± 0.17	1 ± 0.26
	10	1.22 ± 0.13	1.31 ± 0.19	1.36 ± 0.42	4.13 ± 0.88**	1.22 ± 0.23
	100	1.13 ± 0.12	1.33 ± 0.18	0.91 ± 0.18	3.12 ± 0.76**	1.1 ± 0.22
	1,000	1.1 ± 0.28	1.31 ± 0.55	1.06 ± 0.29	3.94 ± 0.66**	0.75 ± 0.16
CCL21	0	1 ± 0.33	1 ± 0.37	1 ± 0.34	1 ± 0.41	ND
	10	1.4 ± 0.28	1.75 ± 0.4	0.96 ± 0.17	1.16 ± 0.12	ND
	100	11.82 ± 1.8***	21 ± 3.32***	2.18 ± 0.25**	2.83 ± 0.63*	ND
	1,000	51.67 ± 5.66***	71.8 ± 7.65****	36.9 ± 4.38***	4.03 ± 2.47	ND
CCL22	0	1 ± 0.57	1 ± 0.69	1 ± 0.55	1 ± 0.56	1 ± 0.09
	10	1.4 ± 0.33	0.7 ± 0.28	0.93 ± 0.61	0.52 ± 0.12	1.06 ± 0.03
	100	0.95 ± 0.38	0.86 ± 0.22	1.47 ± 1.3	0.62 ± 0.02	1.02 ± 0.01
	1,000	1.44 ± 0.9	1.46 ± 0.71	1.66 ± 1.41	1.5 ± 0.99	1.05 ± 0.07
CCL23	0	1 ± 0.25	1 ± 0.27	1 ± 0.24	1 ± 0.23	1 ± 0.14
	10	0.86 ± 0.2	0.85 ± 0.16	0.94 ± 0.24	0.47 ± 0.09	1.04 ± 0.02
	100	0.63 ± 0.06	0.61 ± 0.07	0.65 ± 0.09	0.41 ± 0.18	0.9 ± 0.12
	1,000	0.85 ± 0.09	0.87 ± 0.06	0.93 ± 0.14	0.32 ± 0.14	1.12 ± 0.06
CCL24	0	1 ± 0.57	1 ± 0.69	1 ± 0.55	1 ± 0.56	1 ± 0.09
	10	0.92 ± 0.4	0.5 ± 0.17	1.01 ± 0.74	0.61 ± 0.26	1 ± 0.06
	100	0.77 ± 0.46	0.46 ± 0.24	0.46 ± 0.26	0.33 ± 0	0.97 ± 0.02
	1,000	0.55 ± 0.3	0.46 ± 0.19	0.45 ± 0.23	0.36 ± 0.06	0.89 ± 0.08
CCL25	0	1 ± 0.05	1 ± 0.13	1 ± 0.46	1 ± 0.26	1 ± 0.02
	10	0.69 ± 0.15	0.7 ± 0.23	0.66 ± 0.3	0.58 ± 0.5	0.86 ± 0.06
	100	0.58 ± 0.28	0.58 ± 0.26	1.03 ± 0.65	0.56 ± 0.44	0.64 ± 0.25
	1,000	0.5 ± 0.35	0.68 ± 0.15	1.12 ± 0.91	0.76 ± 0.55	0.73 ± 0.18
CCL26	0	1 ± 0.1	1 ± 0.11	1 ± 0.09	1 ± 0.13	1 ± 0.14
	10	0.47 ± 0.01	0.38 ± 0.04	0.62 ± 0.07	0.67 ± 0.14	0.99 ± 0.18
	100	0.64 ± 0.1	0.46 ± 0.06	0.97 ± 0.36	0.99 ± 0.35	1.11 ± 0.23
	1,000	0.59 ± 0.09	0.5 ± 0.11	0.84 ± 0.08	0.88 ± 0.11	0.94 ± 0.03
CCL27	0	1 ± 0.23	1 ± 0.29	1 ± 0.33	1 ± 0.26	1 ± 0.09
	10	1.34 ± 0.22	1.14 ± 0.08	1.09 ± 0.08	2.03 ± 0.23	1.02 ± 0.07
	100	1.09 ± 0.09	1.14 ± 0.08	0.91 ± 0.03	1.22 ± 0.04	0.92 ± 0.04
	1,000	1.18 ± 0.07	1.41 ± 0.1	0.94 ± 0.03	2.28 ± 0.13*	0.93 ± 0.06
CCL28	0	1 ± 0.57	1 ± 0.69	1 ± 0.55	1 ± 0.56	1 ± 0.09
	10	0.88 ± 0.68	0.71 ± 0.28	0.67 ± 0.5	0.39 ± 0.2	0.84 ± 0.02
	100	0.62 ± 0.28	0.82 ± 0.29	0.8 ± 0.45	0.8 ± 0.33	0.98 ± 0.1
	1,000	0.84 ± 0.39	0.74 ± 0.29	1.69 ± 1.2	1.11 ± 0.68	1.16 ± 0.1
CXCL1	0	1 ± 0.03	1 ± 0.1	1 ± 0.03	1 ± 0.45	1 ± 0.08
	10	1.1 ± 0.08	1.08 ± 0.02	1.09 ± 0.25	1.04 ± 0.19	0.93 ± 0.16
	100	1.02 ± 0.09	1.03 ± 0.1	0.94 ± 0.12	1.42 ± 0.24	0.91 ± 0.19
	1,000	1.01 ± 0.22	0.93 ± 0.19	1.05 ± 0.19	1.23 ± 0.39	0.92 ± 0.2
CXCL2	0	1 ± 0.03	1 ± 0.1	1 ± 0.03	1 ± 0.45	1 ± 0.08
	10	0.97 ± 0.08	0.95 ± 0.08	0.99 ± 0.13	0.73 ± 0.07	0.95 ± 0.23
	100	0.94 ± 0.07	0.92 ± 0.07	0.99 ± 0.25	1.02 ± 0.32	0.86 ± 0.18
	1,000	1.03 ± 0.23	1.02 ± 0.25	0.99 ± 0.25	0.83 ± 0.37	0.96 ± 0.22
CXCL3	0	1 ± 0.05	1 ± 0.13	1 ± 0.46	1 ± 0.26	1 ± 0.02
	10	0.36 ± 0.18	0.57 ± 0.1	0.72 ± 0.54	0.74 ± 0.67	0.92 ± 0.06
	100	0.39 ± 0.29	0.53 ± 0.15	0.97 ± 0.66	1.1 ± 0.89	0.95 ± 0.06
	1,000	0.66 ± 0.51	0.72 ± 0.24	1.05 ± 0.8	0.6 ± 0.51	0.89 ± 0.1
CXCL4	0	1 ± 0.11	1 ± 0.04	1 ± 0.26	1 ± 0.22	1 ± 0.14
	10	0.76 ± 0.34	1.16 ± 0.75	0.53 ± 0.14	0.61 ± 0.33	0.79 ± 0.02
	100	0.71 ± 0.22	1.01 ± 0.09	0.96 ± 0.06	0.38 ± 0.22	0.97 ± 0.11
	1,000	1.51 ± 0.52	1.06 ± 0.54	1.87 ± 0.07*	0.73 ± 0.11	0.95 ± 0.09
CXCL5	0	1 ± 0.04	1 ± 0.08	1 ± 0.01	1 ± 0.17	1 ± 0.31
	10	1.09 ± 0.12	1.56 ± 0.48	1.09 ± 0.17	2.45 ± 0.66	1.15 ± 0.22
	100	1.11 ± 0.08	1.5 ± 0.24	1.44 ± 0.58	2.24 ± 0.31*	0.93 ± 0.21
	1,000	1.32 ± 0.4	1.69 ± 0.73	0.95 ± 0.22	0.38 ± 0.06	1.39 ± 0.26
CXCL6	0	1 ± 0.1	1 ± 0.11	1 ± 0.09	1 ± 0.13	1 ± 0.14
	10	0.53 ± 0.04	0.45 ± 0.06	0.61 ± 0.09	0.97 ± 0.32	0.85 ± 0.11
	100	0.42 ± 0.02	0.36 ± 0.05	0.51 ± 0.13	0.67 ± 0.07	0.95 ± 0.06
	1,000	0.55 ± 0.08	0.44 ± 0.11	0.67 ± 0.08	1.18 ± 0.39	0.96 ± 0.06
CXCL7	0	1 ± 0.05	1 ± 0.13	1 ± 0.46	1 ± 0.26	1 ± 0.02
	10	0.76 ± 0.41	1 ± 0.49	0.57 ± 0.33	0.67 ± 0.46	0.84 ± 0.08
	100	0.59 ± 0.37	0.68 ± 0.24	1.07 ± 0.85	0.76 ± 0.62	0.89 ± 0.08
	1,000	1.05 ± 0.43	1.09 ± 0.03	0.78 ± 0.56	0.73 ± 0.63	0.98 ± 0.01
CXCL8	0	1 ± 0.1	1 ± 0.11	1 ± 0.09	1 ± 0.13	1 ± 0.14
	10	0.5 ± 0.07	0.39 ± 0.05	0.59 ± 0.09	0.87 ± 0.17	0.87 ± 0.07
	100	0.44 ± 0.01	0.32 ± 0.03	0.5 ± 0.03	0.69 ± 0.06	0.83 ± 0.09
	1,000	0.52 ± 0.05	0.47 ± 0.05	0.58 ± 0.05	0.99 ± 0.13	0.9 ± 0.16
CXCL9	0	1 ± 0.17	1 ± 0.15	1 ± 0.16	1 ± 0.18	1 ± 0.23
	10	0.84 ± 0.05	0.96 ± 0.05	0.73 ± 0.05	1.11 ± 0.26	0.9 ± 0.2
	100	0.9 ± 0.07	0.95 ± 0.08	0.73 ± 0.05	0.63 ± 0.1	1.18 ± 0.17
	1,000	3.25 ± 0.22***	2.78 ± 0.43**	2.63 ± 0.01****	1.02 ± 0.3	1.95 ± 0.2**
CXCL10	0	1 ± 0.17	1 ± 0.15	**1 ± 0.16**	1 ± 0.18	1 ± 0.23
	10	4.47 ± 0.19****	2.11 ± 0.19**	5.17 ± 0.16****	1.1 ± 0.19	1.87 ± 0.1**
	100	4.66 ± 0.63***	2.12 ± 0.29**	5.37 ± 0.8***	0.87 ± 0.05	1.7 ± 0.05**
	1,000	4 ± 0.85**	2.1 ± 0.57*	4.21 ± 0.89**	0.84 ± 0.11	1.8 ± 0.18**
CXCL11	0	1 ± 0.44	1 ± 0.41	1 ± 0.5	1 ± 0.35	1 ± 0.25
	10	1.89 ± 0.27*	1.27 ± 0.4	1.93 ± 0.27*	2.2 ± 0.5	1.9 ± 0.26*
	100	6.34 ± 2.17*	3.02 ± 1.24	7.64 ± 2.48*	0.32 ± 0.45	3.2 ± 0.87*
	1,000	1.97 ± 0.7	2.22 ± 0.8	1.76 ± 0.66	1.64 ± 1.03	1.69 ± 0.76
CXCL12	0	1 ± 0.33	1 ± 0.38	1 ± 0.34	1 ± 0.4	1 ± 0.1
	10	2.6 ± 0.44**	2.83 ± 0.52**	2.14 ± 0.35*	3.2 ± 0.39**	1.84 ± 0.19**
	100	6.92 ± 0.28****	8.19 ± 0.34****	5.34 ± 0.38***	9.23 ± 0.85***	3.23 ± 0.35***
	1,000	7.13 ± 0.18****	9.59 ± 0.19****	4.44 ± 0.17****	10.3 ± 0.51****	3.11 ± 0.16***
CXCL13	0	1 ± 0.06	1 ± 0.02	1 ± 0.03	1 ± 0.04	1 ± 0.11
	10	1.19 ± 0.11	1.36 ± 0.15*	1.32 ± 0.04***	2 ± 0.41*	0.93 ± 0.03
	100	1.1 ± 0.04	1.23 ± 0.08**	1.14 ± 0.04**	2.24 ± 0.32**	0.9 ± 0.1
	1,000	1.7 ± 0.02****	2.01 ± 0.04****	2.07 ± 0.13***	27.7 ± 0.47****	1.16 ± 0.06
CXCL14	0	1 ± 0.05	1 ± 0.13	1 ± 0.46	1 ± 0.26	1 ± 0.02
	10	0.53 ± 0.35	0.56 ± 0.18	0.94 ± 0.72	1.09 ± 0.95	1 ± 0.07
	100	0.59 ± 0.34	0.6 ± 0.22	0.73 ± 0.57	1.08 ± 0.73	0.85 ± 0.08
	1,000	0.83 ± 0.6	0.97 ± 0.53	0.92 ± 0.69	0.9 ± 0.55	0.94 ± 0.01
CXCL15	0	1 ± 0.03	1 ± 0.1	1 ± 0.03	1 ± 0.45	1 ± 0.08
	10	0.66 ± 0.1	0.53 ± 0.1	0.8 ± 0.18	1.03 ± 0.19	1.13 ± 0.12
	100	0.47 ± 0.07	0.34 ± 0.01	0.6 ± 0.12	1.03 ± 0.19	0.95 ± 0.14
	1,000	0.63 ± 0.12	0.54 ± 0.12	0.75 ± 0.13	1.04 ± 0.33	1.03 ± 0.2
CXCL16	0	1 ± 0.11	1 ± 0.04	1 ± 0.26	1 ± 0.22	1 ± 0.14
	10	1.34 ± 0.91	0.77 ± 0.37	1.06 ± 0.67	0.84 ± 0.55	0.77 ± 0
	100	1.19 ± 0.7	1.04 ± 0.12	1.1 ± 0.08	0.62 ± 0.46	0.96 ± 0.1
	1,000	1.96 ± 0.07**	0.84 ± 0.31	1.87 ± 0.06*	1.1 ± 0.26	0.92 ± 0.05
CXCL17	0	1 ± 0.38	1 ± 0.47	1 ± 0.32	1 ± 0.39	1 ± 0.23
	10	0.86 ± 0.16	0.84 ± 0.21	0.7 ± 0.16	0.64 ± 0.12	0.98 ± 0.23
	100	0.69 ± 0.19	1.23 ± 0.54	0.67 ± 0.26	0.41 ± 0.12	0.91 ± 0.28
	1,000	1.09 ± 0.41	1.23 ± 0.54	0.77 ± 0.31	0.92 ± 0.4	1.11 ± 0.38
CX3CL1	0	1 ± 0.38	1 ± 0.47	1 ± 0.32	1 ± 0.39	1 ± 0.23
	10	0.9 ± 0.16	0.83 ± 0.17	0.87 ± 0.21	1.01 ± 0.09	1.06 ± 0.14
	100	0.73 ± 0.15	0.61 ± 0.13	0.66 ± 0.21	0.72 ± 0.19	0.91 ± 0.28
	1,000	0.99 ± 0.37	0.94 ± 0.41	0.87 ± 0.3	0.94 ± 0.26	1.05 ± 0.32
XCL1	0	1 ± 0.05	1 ± 0.13	1 ± 0.46	1 ± 0.26	1 ± 0.04
	10	0.61 ± 0.36	0.57 ± 0.16	0.85 ± 0.64	0.79 ± 0.67	0.7 ± 0.24
	100	0.52 ± 0.29	0.47 ± 0.23	0.76 ± 0.55	0.76 ± 0.69	1.04 ± 0.1
	1,000	0.74 ± 0.58	0.62 ± 0.05	1.09 ± 0.77	0.9 ± 0.81	0.92 ± 0.16
XCL2	0	1 ± 0.17	1 ± 0.18	1 ± 0.25	1 ± 0.5	1 ± 0.05
	10	1.62 ± 0.31	1.58 ± 0.27	1.72 ± 0.42	2.83 ± 0.19*	1.04 ± 0.11
	100	1.33 ± 0.22	1.25 ± 0.17	1.58 ± 0.39	1.61 ± 0.01	0.84 ± 0.06
	1,000	1.57 ± 0.01	1.52 ± 0.03	1.74 ± 0.07	2.12 ± 0.4	1.07 ± 0.12
Chemerin	0	1 ± 0.17	1 ± 0.15	1 ± 0.16	1 ± 0.18	0.67 ± 0.47
	10	1.31 ± 0.21	1.22 ± 0.19	1.39 ± 0.23	0.69 ± 0.15	1.42 ± 1.02
	100	1.1 ± 0.21	1.12 ± 0.22	1.07 ± 0.22	0.66 ± 0.06	0.77 ± 0.55
	1,000	1.3 ± 0.33	1.38 ± 0.34	1.3 ± 0.39	0.82 ± 0.35	0.67 ± 0.49

Asterisks indicate a significant difference versus control (0ng/ml) per respective chemokine by unpaired t test: *p < 0.05, **p < 0.01, ***p < 0.001, ****p < 0.0001.

**Figure 2:**
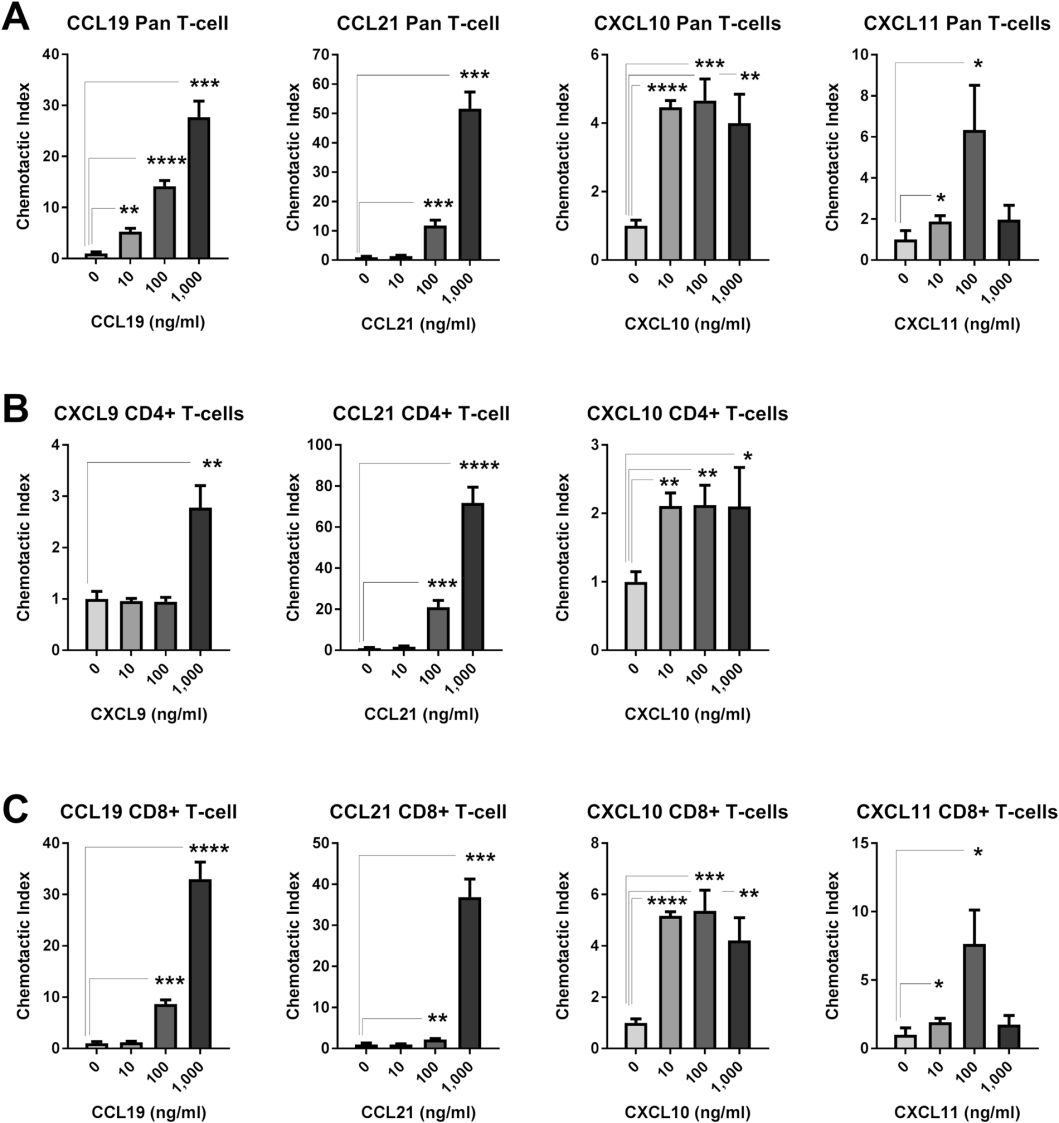
Resting T cell chemoattraction. (**A**) Pan T cell CI in response to CCL19, CCL21, CXCL10, and CXCL11 at the indicated concentration ranges. (**B**) Resting enriched CD4^+^ T cell response to CCL19, CCL21, and CXCL10 at the indicated concentration ranges. (**C**) Resting enriched CD8^+^ T cell response to CCL19, CCL21, CXCL10, and CXCL11 at the indicated concentration ranges. *p < 0.05, **p < 0.01, ***p < 0.001, ****p < 0.0001.

**Figure 3:**
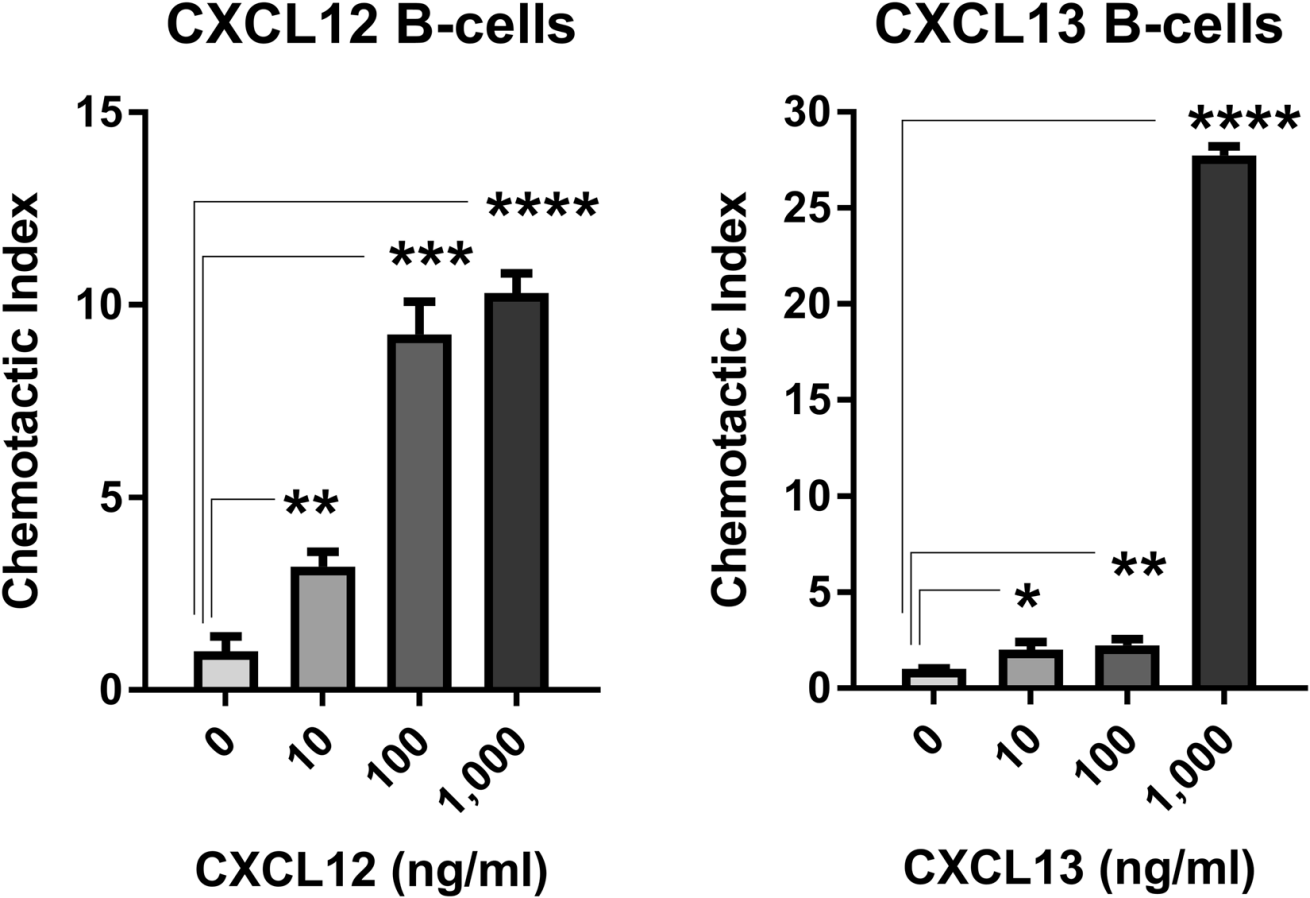
Resting B cell chemoattraction. B cell CI in response to CXCL12 and CXCL13 at the indicated concentration ranges.

**Figure 4:**
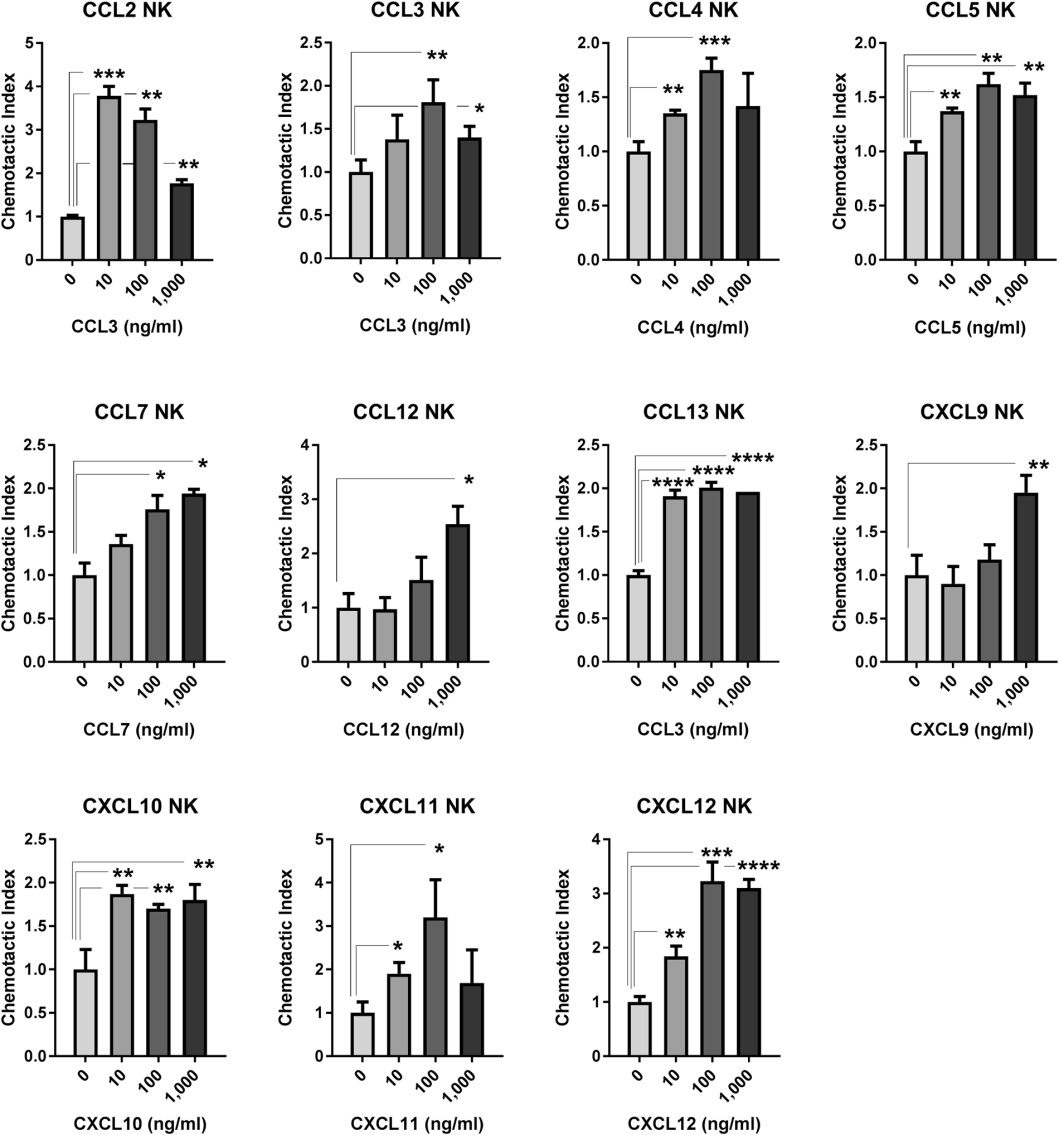
NK cell chemoattraction. NK cell CI In response to CCL2, CCL3, CCL4, CCL5, CCL7, CCL12, CCL13, CXCL9, CXCL10, CXCL11, and CXCL12 at the indicated concentration ranges.

Resting pan T cells displayed significant and concentration dependent chemoattraction toward CCL19 and CCL21, and to a lesser extent CXCL10 and CXCL12. Statistically significant CI was also observed toward CCL16, CXCL9, CXCL11, CXCL13, and CXCL16 at some concentrations, but was of low magnitude or was not concentration-dependent. Resting CD4^+^ and CD8^+^ T cells displayed similar concentration-dependent chemoattraction toward CCL19, CCL21, CXCL10, and CXCL12 as pan T cells, and displayed variable low magnitude CI toward CCL13, CCL16, CXCL9, CXCL11, CXCL13, and/or CXCL16 (Fig. 2 and Table 2). Significant and concentration-dependent chemoattraction of resting B cells was restricted to CXCL12 and CXCL13, with low magnitude statistically significant CI observed toward some concentrations of CCL9, CCL11, CCL12, CCL19, CCL20, CCL21, CCL27, CXCL5, and XCL2 (Fig. 3 and Table 2). Resting NK cells displayed statistically significant and concentration-dependent chemoattraction toward a broad range of chemokines including CCL2, CCL3, CCL4, CCL5, CCL7, CCL12, CCL13, CXCL9, CXCL10, CXCL11, and CXCL12 (Fig. 4 and Table 2). However, NK cells did not display CI values greater than 4 toward any chemokine at any concentration, perhaps reflecting the high baseline chemokinesis displayed by resting NK cells (Fig. 1).

**Figure 5:**
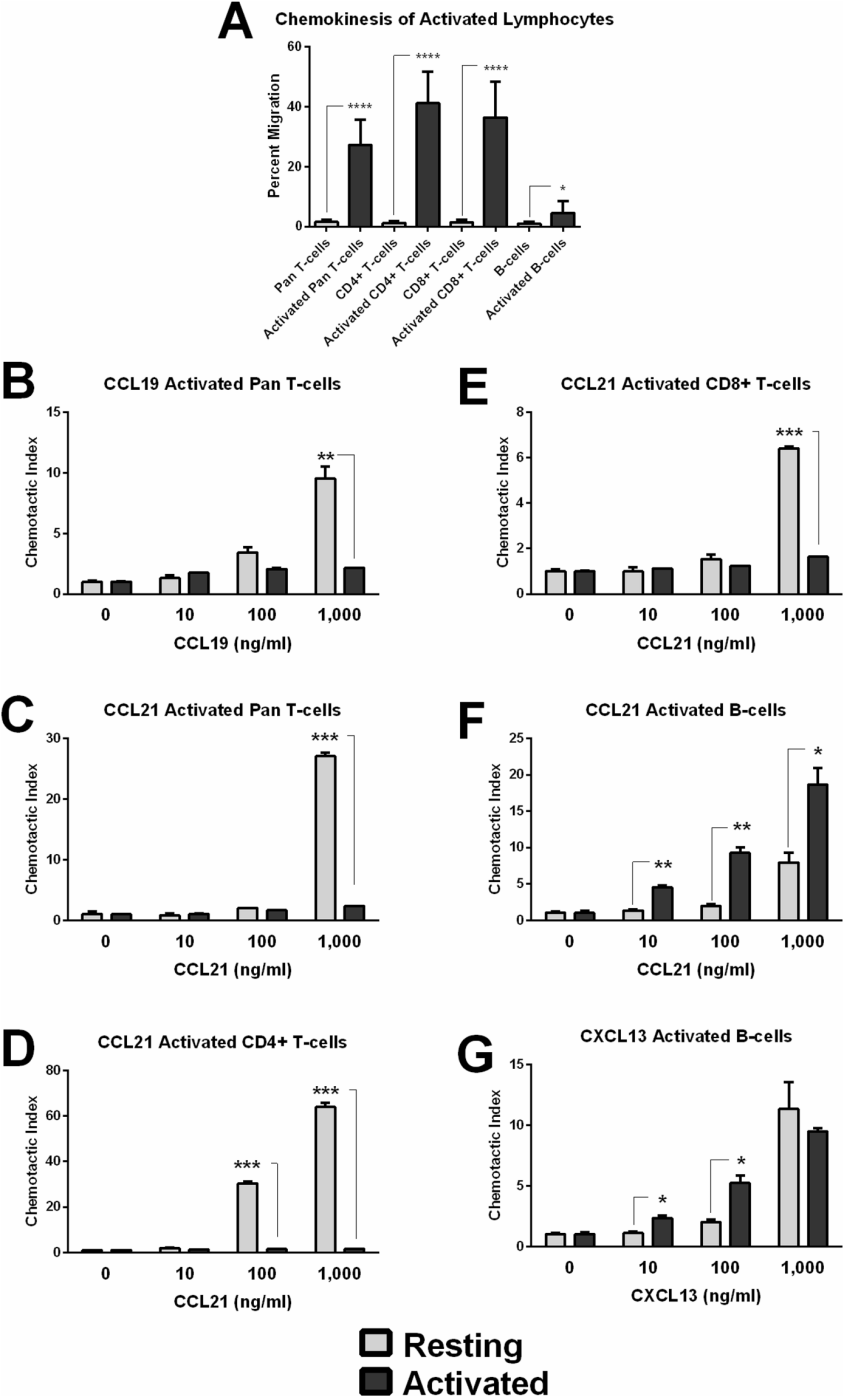
Lymphocyte Activation increases chemokinesis and changes chemotactic response. (**A**) T cell populations activated with plate-bound anti-CD3 and B cells activated with LPS display increased chemokinesis. (**B**) CI of resting or activated pan T cells in response to CCL19. (**C**) CI of resting or activated pan T cells in response to CCL21. (**D**) CI of resting or activated enriched CD4^+^ T cells in response to CCL21. (**E**) CI of resting or activated enriched CD8^+^ T cells in response to CCL21. (**F**) CI of resting or activated B cells in response to CCL21. (**G**) CI of resting or activated B cells in response to CXCL13.

When activated, lymphocytes become more motile and often down-regulate expression of chemokine receptors recognizing secondary lymphoid tissue-homing chemokines such as CCR7. This aids in emigration from the follicle and secondary lymphoid tissues^34^. Here, activation of pan T cells, enriched CD4^+^ T cells, and enriched CD8^+^ T cells with plate-bound anti-CD3 resulted in significantly increased chemokinesis. Similarly, but to a lesser extent, activation of B cells with LPS significantly increased chemokinesis (Fig. 5A). Despite higher baseline motility, activated T cells loose chemoattraction to lymphoid-homing chemokines as demonstrated by reduced CI toward CCL19 and CCL21 for pan T cell populations (Fig. 5B,C), and reduced chemoattraction of enriched CD4^+^ and CD8^+^ T cells toward CCL21 (Fig. 5D,E). In contrast, B cells displayed significantly increased chemoattraction toward CCL21 and CXCL13 upon activation with LPS (Fig. 5F,G). This likely reflects the divergent roles of B cells and T cells following activation in secondary lymphoid tissues with T cells often emigrating T-cell zones and recirculating into the periphery and with B cells clustering in follicles.

The standardized set of chemotaxis assays presented here demonstrate that chemokines differentially affect random and directed motion of lymphocytes. To investigate the implications of this regarding immune response and subsequent tumor growth depending on the presence of the ELNs we developed a phenomenological mathematical model of the tumor and surrounding environment that is able to make use of chemoattraction data an simulate different scenarios.

**Figure 6:**
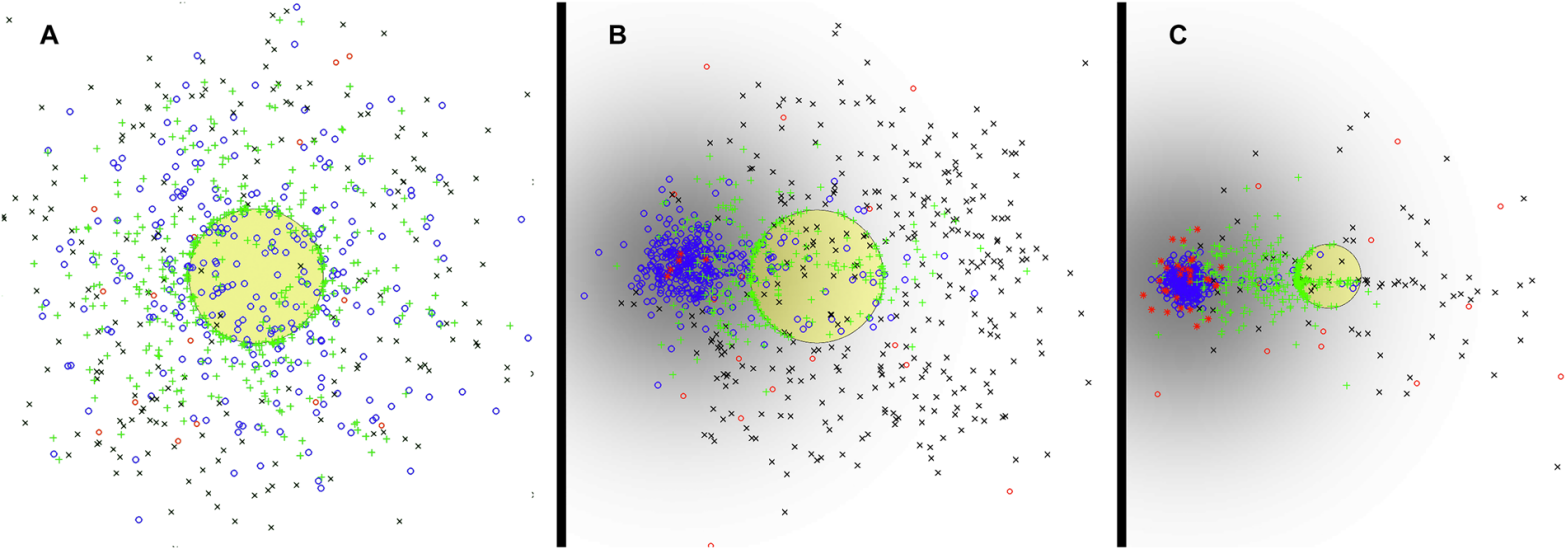
Snapshots of the mathematical model on day 14 of the simulation. (**A**) No RFC cells in the simulation. Tumor is in yellow. APC^OFF^ (red circles), APC^M^ (blue circles), inactive T cells (black ‘x’) and activated T cells (green ‘ + ’) are shown. (**B**) 5 RFC cells (red stars). Normalized chemokine gradient shown in gray. (**C**) 30 RFC cells.

**Figure 7:**
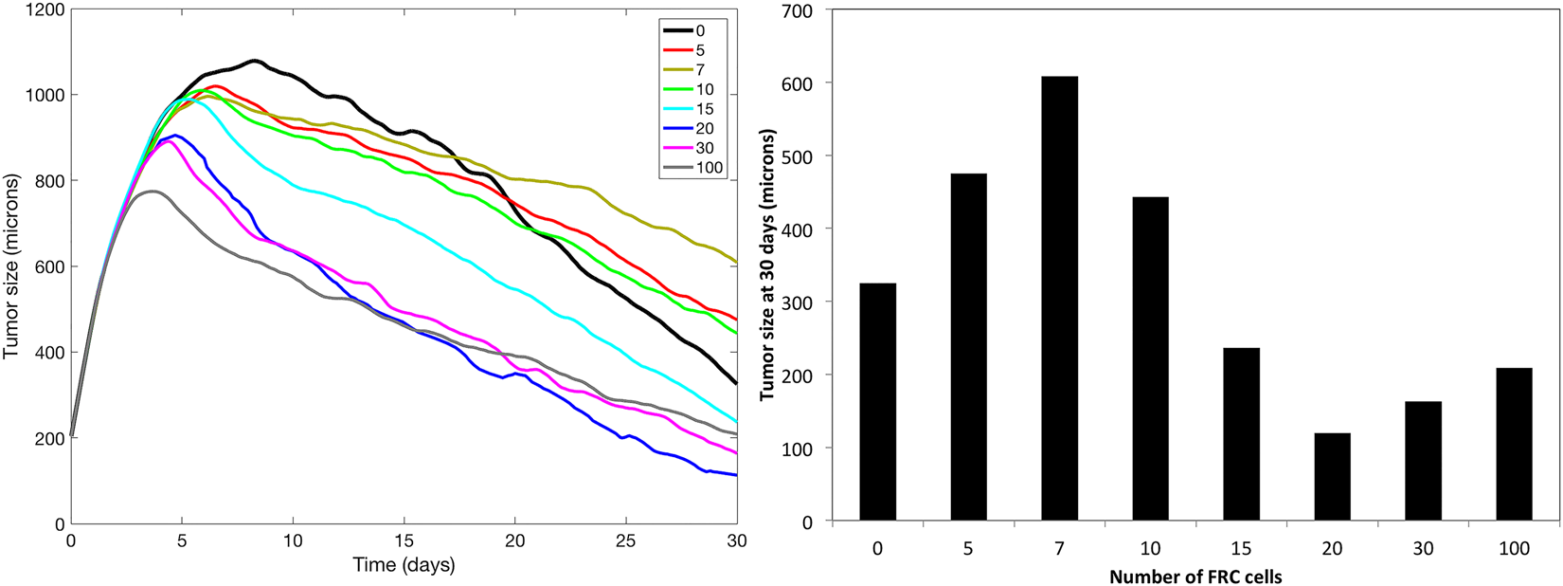
Left: Tumor dynamics for different numbers of RFC (from 0 to 100) in the simulation. All plots are the average of 5 runs. Right: Tumor size at 30 days for each simulation.

Figure 6 shows snapshots of the model at 14 days of simulation, under three different conditions for the RFC: (a) no RFC, (b) 5 RFC, and (c) 30 RFC. Time course plots for these and several other simulations with different RFC counts are shown in Fig. 7, in the left panel. The results suggest that the presence of the RFC has an impact on the immune response to the tumor, both in terms of T-cell activation and tumor reduction. In all cases, the tumor grows for an initial period since the immune system starts in an inactive state. Eventually, APCs present tumor antigens to inactive T cells, causing activation, migration, and accumulation of TILs inside the tumor and eventual tumor regression. The presence of higher numbers of RFC causes a faster immune response to occur: without an ELNs, the peak tumor size occurs at day 9; while for simulations with 100 RFC the peak tumor size is around 4 days, suggesting that the migratory organization of lymphocytes provided by the ELNs significantly accelerates the anti-tumor immune response. In the case with 100 RFC, the tumor also reaches a maximum size that is about 25% smaller than the simulation with no RFC. This advantage persists as tumor growth continues to decline. The right panel of Fig. 7 compares tumor sizes after 30 days of simulation. Of interest, having a small number of RFC cells (5–10) does not produce an immune response as effective as the case with no RFC or a high number of RFC. This nonlinear result is because the weak chemokine signal of the ELNs draws some nearby cells away from the tumor, but a lack of immune cell density limits the effectiveness of rapid activation. In essence, there is a need for critical mass in the ELNs to achieve efficient cell attraction and activation. This can be seen in Fig. 6B, where the dendritic cells are accumulated in the ELNs area, but inactive T cells remain predominantly in the tumor microenvironment due to the weak signaling from the ELNs patch.

## Discussion

We had earlier identified a unique tumor-derived, 12-chemokine gene expression signature that could accurately predict the degree and type of lymphoid infiltrate, organized remarkably as ELNs that comprise - by immunohistochemistry staining - prominent B cell follicles, T cell marginal zones comprised of both CD4^+^ and CD8^+^ cell subsets, and associated follicular dendritic cells^23^. Of importance, there was a highly significant and consistent association between a marked increase in overall patient survival, the value of the mean score of this gene expression signature, and the presence of ELNs in stage IV (non-locoregional) melanoma, colorectal cancer, and stage IV bladder cancer. However, we found that the majority of human solid tumors lacked the presence of these particular ELNs, and patients harboring these ‘poorly-immunogenic’ tumors have had uniformly poor prognosis (i.e. reduced overall survival). Thus, there is a clear unmet medical need to ‘reverse’ this tumor microenvironment (that lacks these particular ELNs) by manufacturing *de novo* ‘designer’ ELNs.

Lymphocyte recruitment to the tumor microenvironment represents an attractive target to enhance anti-tumor immunity. As we continue to study lymphocyte trafficking, it is increasingly necessary to view chemokine-mediated trafficking as networks of chemokines and chemokine receptors working in concert as opposed to individual chemoattractive axes. Understanding how chemokine receptor expression and lymphocyte responsiveness changes during the course of maturation, activation, and effector response will greatly inform the development of preclinical animal and mathematical models. The chemokine database described herein may serve as a normalized resource to parameterize such models.

Given a direct link between gene expression of certain chemokines within human solid tumor microenvironments, the presence of tumor-localized ELNs, and addressing a clinical unmet need, we embarked on developing a standardized database of chemoattractive potentials of immune cell subsets for a broad range of chemokines to identify candidates for future engineering of ELNs. To approach this goal, we developed a strategy that combined the use of defined, recombinant chemokines, highly enriched resting and activated immune cell subsets, with standardized microchemotaxis assays to provide potential leads to further test in mathematical models. In this regard, we are developing both mathematical and pre-clinical animal models of ELNs formation in which multiple elements are being interrogated. The inclusion of lymph node-derived primary cellular components, which normally provide chemotactic and homeostatic queues in conventional lymph nodes, are being genetically modified to express selected chemotactic (e.g., see Table 3) and lymphoid neogenesis-related genes to enhance ELN formation. These modified cell lines are being combined with tumor antigen-pulsed dendritic cells and then incorporated in biocompatible scaffold materials and administered to tumor-bearing mice as injectable or implantable matrices^35^. These matrices may provide two potential benefits when delivered to tumor-bearing hosts. First, they may serve as model systems to better understand the factors governing the formation and/or maintenance of ELNs with anti-tumor reactivity. Second, these matrix-based systems may function as a therapeutic platform by delivering, stimulating and expanding transplanted lymphocytes and/or dendritic cells. In addition, the inert nature of bio-scaffolds also allows for the implementation of microparticle or nanoparticle constructs for controlled release of soluble factors. Such measures can provide sustained environmental queues to augment antigen-presenting cell or lymphocyte longevity, maturation, and activation. The initial mathematical modeling presented herein will be further developed to include these parameters.

**Table 3:** Chemotactic Index (CI) Summary for the 12-chemokine GES on Resting Lymphocytes.

Cell Type	Pan T	CD4+T	CD8+T	B	NK
CCL2	**—**	**—**	**—**	**—**	**−/ + **
CCL3	**—**	**—**	**—**	**—**	**−/+**
CCL4	**—**	**—**	**—**	**—**	**−/+**
CCL5	**—**	**—**	**—**	**—**	**−/+**
CCL8	**—**	**—**	**—**	**—**	**—**
CCL18	—	**—**	**—**	**—**	**—**
CCL19	**+**	**+**	**+**	**−/+**	**ND**
CCL21	**+**	**+**	**+**	**−/+**	**ND**
CXCL9	**−/+**	**−/+**	**−/+**	**—**	**−/+**
CXCL10	**−/+**	**−/+**	**+**	**—**	**−/+**
CXCL11	**+**	**—**	**+**	**—**	**−/+**
CXCL13	**−/+**	**−/+**	**−/+**	**+**	**—**

“**−**” No significant difference in CI at any chemokine dose.

“**−**/+” p < 0.05 for at least one chemokine dose, but CI less than 5 for at least one chemokine dose.

“+” p < 0.05 for at least one chemokine dose, and CI over 5 for at least one chemokine dose lg.

ND Not determined.

In regard to translating *in vitro* migration assays to *in silico* models, care must be taken to differentiate between non-directional movement known as chemokinesis and directional chemotaxis toward a chemokine gradient. CI, as it is calculated here, normalizes transwell migration relative to baseline chemokinesis. This denominator is greatly increased after lymphocyte activation. Without normalizing to baseline movement, the percentage of cells migrating may appear very high when the majority of migration can be accounted for by random motility. Because this database was constructed using a single methodology, time point, and concentration range, CI can be compared across chemokines. For example, CCL19 and CCL21 are both well studied T cell chemoattractants; however, here CCL21 appears to be a 50% stronger chemoattractant compared to an equimolar amount of CCL19. In addition, this database serves to point out suboptimal chemoattractants whose combined cumulative effects may play an important role for *in silico* models such as the broad responsiveness observed for NK cells.

As a first step toward integrating this type of chemotactic data into an *in silico* model of ELNs formation, we developed a simple phenomenological mathematical model that predicts the chemokine gradient created as a result of lymphocyte, APC, and stromal interactions in the tumor microenvironment. Here, we consider secretion of a general chemoattractant for responding lymphocytes, and can serve as the basic groundwork for simulating multiple chemokine/chemoattraction axes involving more detailed cellular phenotypes. Further investigation of relevant chemokine relations between APC, RFC, and T cells would lead to a more mechanistic implementation of the model, which could then inform the design of future *in vivo* studies. The generalized gradient presented here can be replaced by specific chemokines observed in the microenvironment of tumor samples, or with chemokines that are known to be secreted by activated DC and/or stroma. In this way, multiple gradients can be modeled in concert. In addition, each chemokine gradient can be further developed beyond a two-dimensional Gaussian distribution taking into account extracellular matrix components that can bind to and slow the degradation of chemokines, better representing lymphocyte conduit systems observed in follicular structures *in vivo* ^28^. So too can the responsiveness of activated cell populations be adjusted based on lymphocyte activation, and on negative feedback mediated by shingosine-1-phosphate receptor (S1P_1_) activity^36–38^.

Importantly, this model outlines the framework to simulate chemotactic movement of infiltrating populations in response to chemokine gradients. This alone inadequately represents the physiologic tumor microenvironment, and in particular does not address immune cell polarity or immunosuppression. In addition to representing more defined subpopulations of immune cells, other soluble factors governing tumor-induced immune suppression will likely need to be added to fully capture the dynamic interplay of pro and anti-tumor elements. In particular, TFG-β, IL-10, and regulatory T cell populations are prevalent components of the tumor microenvironment known to suppress anti-tumor immunity^39^. Concurrently, the production of type I-polarizing factors such as IFN-γ and IL-12 can also be added as a counterbalance to these type II-polarizing factors^40^. Furthermore, it is now appreciated that the tumor as a system is heterogeneous, composed of numerous dominant and subdominant clones with related but different genetic lesions and mutational loads^46^. This diversity results in compartmentalized spatial fields across the tumor bead, each with its own variation of environmental queues, grow rates, and immune involvement^47^. It is reasonable to expect that variations in the type and amount of soluble factors released can also vary across the tumor bed in accordance with clonal diversity. Such variations would directly impact the localization and function of infiltrating immune cells, and may be required to fully model the complex tumor physiology.

The addition of these factors in future models would result in a system whose outcome is not guaranteed, but would rather depend on the balance of pro and anti-tumor forces recruited or induced in the tumor microenvironment. The complexity such models will increase exponentially with the addition of individual chemokine gradients, further subdivision of inflammatory or suppressive cell types, the addition of polarizing soluble factors, and representation of clonal heterogeneity, necessitating the use of standardized datasets such as is presented here. The model introduced here, though simplified, clearly demonstrates the potential benefit of ELNs and suggests that stromal/APC interaction is paramount for effector lymphocyte organization and response. Continued development of integrated ELNs formation models will greatly inform potential points of therapeutic intervention, and generate novel hypotheses regarding anti-tumor immunity.

## Electronic supplementary material

Supplementary Information
