# Supplementary material for: Systematic Screening of Chemokines to Identify Candidates to Model and Create Ectopic Lymph Node Structures for Cancer Immunotherapy
Source: Sci Rep. 2017 Nov 22;7:15996. doi: 10.1038/s41598-017-15924-2 (PMC5700067; doi:10.1038/s41598-017-15924-2)
Supplement: Supplementary file 1 — Supplementary Information [file 41598_2017_15924_MOESM1_ESM.doc]

Supplemental Information:

**Systematic Screening of Chemokines to Identify Candidates to Model and Create Ectopic Lymph Node Structures for Cancer Immunotherapy**

Yohsuke Yagawa^a^, Mark Robertson-Tessi^b^, Susan L. Zhou^b^, Alexander R. A. Anderson^b^, James J. Mulé^†a,c^, and Adam W. Mailloux^†a*^,

^a^Department of Immunology, Moffitt Cancer Center, 12902 Magnolia Drive, Tampa, FL 33612

^b^Department of Integrated Mathematical Oncology, Moffitt Cancer Center, 12902 Magnolia Drive, Tampa, FL 33612

^c^Cutaneous Oncology Program, Moffitt Cancer Center, 12902 Magnolia Drive, Tampa, FL 33612

Corresponding Author:

Adam Mailloux, PhD

Immunology Program

H. Lee Moffitt Cancer Center

12592 Magnolia Dr.

Tampa, FL, 33612

Adam.mailloux@moffitt.org

813-754-8229

Supplemental Information: 3 Figures

**Key Words**: Ectopic Lymph Node Structure, Tertiary Lymph Node Structure, Mathematical Model, Chemokines, Cancer, Melanoma, Lymphocyte, Antigen Presenting Cell.

**Conflicts of Interests:** Authors have no conflicts of interest to disclose.

Supplemental Figure 1. Enrichment purities following immunomagnetic isolation of cell populations.

Supplemental Figure 2. Optimization of pore size for chemotaxis assay.

Supplemental Figure 3. Activation of Lymphocytes. (A) Increase in cell size for activated T cells (left) and B cells (right). (B) Increase in forward and side-scatter characteristics for activated T cells (left) and B cells (right). (C) Confirmation of activation markers for T cells (left; CD25; blue) and B cells (right; CD69; blue) versus negative staining controls (red dashed line). (D) Interferon gamma (IFNγ) production by activated T cells (left) and increased IgM production by activated B cells (right) as measured by ELISA.
